# Paromomycin improves clinical outcomes and growth performance compared with halofuginone in dairy calves naturally exposed to *Cryptosporidium parvum*

**DOI:** 10.1016/j.vas.2026.100828

**Published:** 2026-08-18

**Authors:** B.P. Santarosa, M.P.D.F. Branco, G.L. Marques, K.N. da Silva, A.L. Lelis, G.M. Januario, G.G. Silveira, R.E. Lopes

**Affiliations:** a HUVEPHARMA, Av. Borges de Medeiros, 2500 - Conj 1604 1605 - Praia de Belas, Porto Alegre, RS, 90110-150, Brazil; b School of Veterinary Medicine and Animal Science (FMVZ), University of São Paulo (USP), São Paulo, São Paulo, Brazil; c Federal University of the Jequitinhonha and Mucuri Valleys (UFVJM), Unaí, Minas Gerais state, Brazil; d HUVEPHARMA, Uitbreidingstraat 80, Antwerpen, 2600, Belgium

**Keywords:** Dairy calves, Dehydration, Neonatal diarrhea, Protozoal enteropathogens

## Abstract

Neonatal cryptosporidiosis is a major cause of diarrhea, impaired growth, and economic losses in dairy calves. This study compared the effects of halofuginone and paromomycin on diarrhea occurrence, oocyst shedding, and growth performance in preweaned Holstein calves naturally exposed to *Cryptosporidium parvum*. Seventy newborn calves were randomly allocated to receive halofuginone (n = 30; 100 µg/kg body weight) or paromomycin (n = 40; 50 mg/kg body weight) orally for seven consecutive days from 5 to 11 days of age. Diarrhea incidence, duration, hydration requirements, body weight development, and fecal oocyst excretion were monitored longitudinally. Calves treated with halofuginone had significantly higher odds of diarrhea compared with paromomycin-treated calves (OR = 4.93; 95% CI: 2.98–8.17; P < 0.0001). Halofuginone treatment was also associated with longer diarrhea duration (RR = 3.40; 95% CI: 2.20–5.24; P < 0.0001) and more days requiring hydration therapy (RR = 1.93; 95% CI: 1.48–2.50; P < 0.0001). From the second week of life onward, paromomycin-treated calves exhibited greater body weight, with significant differences observed during the preweaning period. Fecal oocyst shedding showed a pronounced peak in halofuginone-treated calves, whereas paromomycin-treated calves displayed a more stable and controlled excretion pattern. These findings indicate that paromomycin provides improved clinical control of neonatal diarrhea and supports better early-life growth compared with halofuginone in dairy calves naturally exposed to *C. parvum*.

## Highlights

• Paromomycin reduced diarrhea incidence and duration in calves.
• Halofuginone increased the odds of diarrhea nearly fivefold.
• Paromomycin improved early-life body weight gain.
• Oocyst shedding was more stable with paromomycin.
• Hydration needs and clinical severity were lower with paromomycin.

## Introduction

Neonatal diarrhea is one of the most important diseases affecting preweaned dairy calves worldwide and may result from infectious or non-infectious causes. Infectious diarrhea includes viral, bacterial, and parasitic etiologies, whereas non-infectious diarrhea is commonly associated with nutritional, environmental, or management-related factors. According to the Dairy Calf and Heifer Association (DCHA) Gold Standards (2026), diarrhea (scours) and bovine respiratory disease are the two most frequently observed health disorders in young calves and remain the leading contributors to morbidity and mortality during the preweaning period. The DCHA recommends a target incidence risk of <25% for mild preweaning diarrhea and <20% for preweaning pneumonia, emphasizing their major impact on calf health and future productivity. Globally, neonatal diarrhea affects approximately 14–39% of dairy calves and is associated with substantial economic losses resulting from treatment costs, labor, reduced average daily gain, delayed age at first calving, and lower milk production in the first lactation (Abuelo et al., 2021; Bernal-Córdoba et al., 2025; Boulton et al., 2017; Roblin et al., 2023). Among infectious causes, *Cryptosporidium parvum* is recognized as one of the most important enteropathogens affecting calves during the first weeks of life. Consequently, preventive strategies targeting cryptosporidiosis have become a priority in commercial dairy operations, particularly using halofuginone and paromomycin, the two compounds with the most consistent evidence of anticryptosporidial activity under field conditions.

*Cryptosporidium parvum* is a major cause of neonatal diarrhea in calves less than three weeks of age. Infection occurs through ingestion of oocysts, and the parasite’s intracellular but extracytoplasmic life cycle enables rapid multiplication and shedding, with infected calves potentially excreting up to 10¹⁰ oocysts per day, contributing to environmental contamination and zoonotic dissemination (Adkins, 2022; Thomson et al., 2017). In Brazil, the average prevalence of *Cryptosporidium* spp. in cattle has been estimated at 30.5% (Oliveira et al., 2023). Because *Cryptosporidium* spp. exhibit limited susceptibility to conventional anticoccidial compounds used against Eimeria spp. and other coccidia (Shahiduzzaman & Daugschies, 2012), halofuginone has historically been the primary prophylactic option available for calf cryptosporidiosis. Halofuginone, a synthetic derivative of febrifugine, exerts its antiparasitic activity through inhibition of aminoacyl-tRNA synthetases, impairing protein synthesis in *C. parvum* (Gill & Sharma, 2022). A meta-analysis of 14 studies demonstrated that prophylactic administration of halofuginone reduces oocyst shedding and delays clinical disease (Silverlås et al., 2009). However, concerns regarding toxicity, anorexia, intestinal irritation, reduced milk intake, and inconsistent effects on growth performance have limited its use under certain field conditions (Almawly et al., 2013; Bernal-Córdoba et al., 2025; Brainard et al., 2020; Niine et al., 2018; Trotz-Williams et al., 2011).

Paromomycin, an aminoglycoside antibiotic with anticryptosporidial activity, has emerged as a promising alternative for the prevention and control of neonatal cryptosporidiosis. Previous studies demonstrated that paromomycin reduces oocyst shedding, decreases diarrhea duration, and exerts minimal adverse effects on feed intake and intestinal health (Bellosa et al., 2011; Fayer & Ellis, 1993; Grinberg et al., 2002). More recently, Vázquez-Flores et al. (2024) reported that prophylactic administration of paromomycin (50 mg/kg for seven consecutive days) reduced diarrhea incidence and *Cryptosporidium* sp. oocyst excretion while improving body weight gain at 60, 90, and 120 days of age compared with calves receiving halofuginone. These findings suggest that paromomycin may provide additional benefits beyond parasite control, particularly regarding calf growth and overall health performance.

Despite the availability of both compounds, direct comparisons between halofuginone and paromomycin under commercial dairy farm conditions remain limited, particularly in tropical production systems characterized by high pathogen pressure and management variability. Furthermore, most previous studies have focused primarily on oocyst shedding or short-term clinical outcomes, with less emphasis on growth performance and integrated health responses. Because halofuginone is widely recognized as the reference prophylactic treatment for bovine cryptosporidiosis and withholding preventive intervention under natural exposure conditions could raise ethical and welfare concerns, the present study was designed to compare paromomycin directly with halofuginone rather than include an untreated control group. We hypothesized that paromomycin would reduce diarrhea frequency and oocyst shedding to a similar or greater extent than halofuginone while promoting improved growth performance. Therefore, the objective of this study was to compare the effects of halofuginone and paromomycin on diarrhea occurrence, *Cryptosporidium parvum* oocyst shedding, and growth performance in preweaned Holstein calves naturally exposed to *C. parvum*.

## Materials and methods

### Animals and experimental design

The experiment was conducted at Fazenda Santa Rita (Agrindus S/A, Descalvado, São Paulo, Brazil) from July to October 2025. Seventy newborn Holstein calves were randomly allocated to 2 treatment groups: halofuginone (n = 30; commercial formulation Halocur®, MSD, 0.5 mg/mL; 100 µg/kg BW) and paromomycin (n = 40; commercial formulation Parofor® Oral Solution, Huvepharma, 140 mg/mL; 50 mg/kg BW). Treatments were administered orally for 7 consecutive days, from d 5 to d 11 of age, approximately 40 min after the morning milk feeding. An unequal allocation ratio (40 paromomycin-treated calves and 30 halofuginone-treated calves) was established before study initiation to increase the amount of efficacy and safety data generated for paromomycin under commercial conditions characterized by a high challenge of neonatal cryptosporidiosis. Maternal parity was recorded at enrollment and categorized as first-parity dams (heifers approaching their first calving) or multiparous dams (cows approaching their second or subsequent calving). For calves resulting from embryo transfer, the parity of the recipient dam that carried the pregnancy and delivered the calf was considered for the analysis. Calves were allocated to treatment groups by simple randomization at enrollment, and maternal parity was not used as a stratification factor during randomization.

Inclusion criteria included eutocic calving, good vitality at birth, body weight greater than 25 kg, and adequate passive immune transfer. Colostrum quality was assessed using a Brix refractometer, and passive immunity transfer was confirmed at 48 h of age by Brix serum. Failure of passive transfer of immunity was defined as a serum Brix value ≤ 8.1%, according to Godden et al. (2019).

Calves were housed individually in suspended hutches with straw bedding and provided free access to water and a commercial starter concentrate (22% crude protein). Whole milk (14% total solids) was offered twice daily via bottle feeding until 68 d of age, following the feeding schedule: 6 L/d from birth to d 12, 8 L/d from d 13 to 50, 6 L/d from d 51 to 60, and 4 L/d from d 61 to 68. From d 69 to 75, milk allowance was reduced to 2 L once daily before weaning.

### Clinical and growth monitoring

Physical examinations included rectal temperature (°C), with fever defined as values > 39.4 °C, and assessment of hydration status (Smith, 2009). Fecal consistency was visually scored at the time of fecal sample collection on the 5th day of life (D1, corresponding to the first day of treatment), as well as on the 7th, 9th, 11th, 19th, and 26th days of age, according to the scoring system proposed by McGuirk (2008): 0 = normal, firm, brown feces with a clean and dry perineum and tail; 1= soft, semi-formed feces; 2 = loose feces with increased water content and adherence of fecal material to the perineum and tail; and 3= watery feces with marked adherence of fecal material to the perineum and tail. Calves presenting fecal scores of 2 or 3 were classified as positive for neonatal diarrhea and received 2 L of oral electrolyte solution administered via bottle feeding, following standard clinical support protocols (Ribeiro Filho et al., 2021). Growth was monitored by estimating body weight at predefined time points, and morphometric measurements, including withers height and hip width, were measured using a measuring tape at birth and at weaning.

### Parasitological analysis

Fecal samples for *Cryptosporidium* sp. oocyst counting were collected from all calves by gentle rectal stimulation (Connor et al., 2017) and stored in 50 mL conical tubes at D1 (5th day of life; immediately before treatment initiation, baseline), D3 (7th day of life; third day of treatment), D5 (9th day of life; fifth day of treatment), D7 (11th day of life; last day of treatment), and D14 (19th day of life; 7 days after the end of treatment) concurrently with examination of fecal consistency. Aliquots of 4 g of feces were transferred in duplicate to 15 mL tubes containing 2.5% potassium dichromate solution and stored under refrigeration to preserve oocyst viability (Arrowood, 2020).

In the laboratory, purification procedures followed Adeyemo et al. (2019) and Arrowood (2020). Samples were vortexed, and 5 mL of suspension were transferred to 15-mL Falcon tubes and filtered through gauze to remove debris. The volume was completed to 10 mL with distilled water and centrifuged at 2000 × *g* for 15 min to remove potassium dichromate. The supernatant was discarded, and the procedure was repeated three times until the supernatant was colorless.

Lipid debris was removed by adding 3 mL of diethyl ether, followed by vigorous shaking and centrifugation (2000 × g for 15 min). The supernatant was removed with a Pasteur pipette, retaining the aqueous sediment containing oocysts.

Oocyst flotation was performed using a sucrose solution (density 1.205 g/mL) according to Ichikawa-Seki et al. (2019) and Adeyemo et al. (2019). The sediment was resuspended in 10 mL of sucrose solution and centrifuged (2000 × g for 15 min). Approximately 500 µL of distilled water were gently added at the interface between the sucrose and the sediment, and oocysts were transferred using a sterile bacteriological loop. The aqueous phase was collected with a Pasteur pipette and transferred to a new 15-mL tube. This process was repeated twice, yielding a final volume of approximately 1.5 mL.

The concentrated material was diluted to 10 mL with distilled water, vortexed, and centrifuged again (2000 × g for 15 min). The supernatant was discarded, and the pellet was resuspended in 1 mL of distilled water and vortexed. Oocyst counting was performed in a Neubauer chamber under a light microscope at 400× magnification (Adkins, 2022; Ichikawa-Seki et al., 2019).

The number of oocysts per gram of feces (OPG) was calculated using the following formula: OPG = [ (number of oocysts counted) × 50,000 × Vfinal]/ m feces, where 50,000 is the Neubauer chamber correction factor, Vfinal​ is the final suspension volume (mL), and m feces is the fecal mass (g) processed.

Quantitative analyses were performed by the GeCria research group at the Department of Veterinary Clinical Medicine, School of Veterinary Medicine and Animal Science, University of São Paulo (FMVZ-USP, São Paulo, Brazil).

### Statistical analysis

Data were analyzed using SAS (version 9.0; SAS Institute Inc., Cary, NC), and figures were generated using R (version 4.3.1). Calf was considered the experimental unit, and statistical significance was set at *P* ≤ 0.05. Daily diarrhea incidence (fecal score ≥ 2; yes/no) and the occurrence of fever were analyzed using generalized estimating equations (GEE) with a binomial distribution and logit link to account for repeated measurements within calves. Treatment, day of life, and their interaction were included as fixed effects. The total number of days with diarrhea and the number of days requiring hydration were analyzed using negative binomial regression models with treatment as the fixed effect.

Colostrum Brix, serum Brix at 48 h, and fecal oocyst counts were evaluated using descriptive statistics (PROC MEANS) and tested for normality using the Shapiro–Wilk test. Colostrum Brix values were tested for normality using the Shapiro–Wilk test. Because normality assumptions were not met, comparisons between primiparous and multiparous cows within the paromomycin group were performed using the Mann–Whitney U test. Variables that did not meet normality assumptions were log10-transformed and subsequently analyzed using linear mixed-effects models (PROC MIXED), with treatment included as a fixed effect and day as a random effect. The use of hydration treatment was analyzed using logistic regression models, and results were expressed as odds ratios (OR) with corresponding 95% confidence intervals. Body weight and skeletal development variables (withers height and rump width) were analyzed using linear mixed-effects models including treatment, time, and their interaction as fixed effects, with calf specified as a random effect. Least squares mean was estimated, and treatment effects within time were evaluated when significant interactions were detected.

## Results

### Inclusion criteria

Colostrum quality and passive transfer of immunity did not differ between treatment groups. As Brix data were not normally distributed, results are presented as median (IQR); however, means and standard deviations are also provided for descriptive purposes. Median colostrum Brix values were 30.1% (IQR: 28.0–31.3) in the paromomycin group and 28.5% (IQR: 27.1–30.5) in the halofuginone group (P = 0.226), corresponding to mean ± SD values of 30.0 ± 3.1% and 29.1 ± 2.5%, respectively (P = 0.193). Likewise, serum Brix values at 48 h were similar between groups, with median values of 10.7% (IQR: 10.0–11.5) and 10.5% (IQR: 9.9–11.2) for the paromomycin and halofuginone groups, respectively (P = 0.447). Corresponding mean ± SD values were 10.8 ± 1.0% and 10.4 ± 1.2%, respectively (P = 0.160). These findings indicate comparable colostrum quality and passive transfer status at enrollment.

The distribution of dam parity, classified as first-parity or multiparous, did not differ between treatment groups (Fisher's exact test, P = 0.130). In the halofuginone group, all dams were multiparous (30/30; 100.0%), whereas in the paromomycin group, 4 of 40 dams (10.0%) were first-parity and 36 (90.0%) were multiparous. Of the 70 calves enrolled in the study, 12 (17.1%) resulted from embryo transfer, including 6 of 30 calves (20.0%) in the halofuginone group and 6 of 40 calves (15.0%) in the paromomycin group. For these calves, parity was determined based on the recipient dam. Within the paromomycin group, colostrum Brix did not differ between first-parity dams (n = 4; median, 27.35%; IQR, 25.93–29.48%) and multiparous dams (n = 36; median, 30.30%; IQR, 28.15–31.20%; Mann–Whitney test, P = 0.250).

### Diarrhea incidence over time

**Fig. 1:**
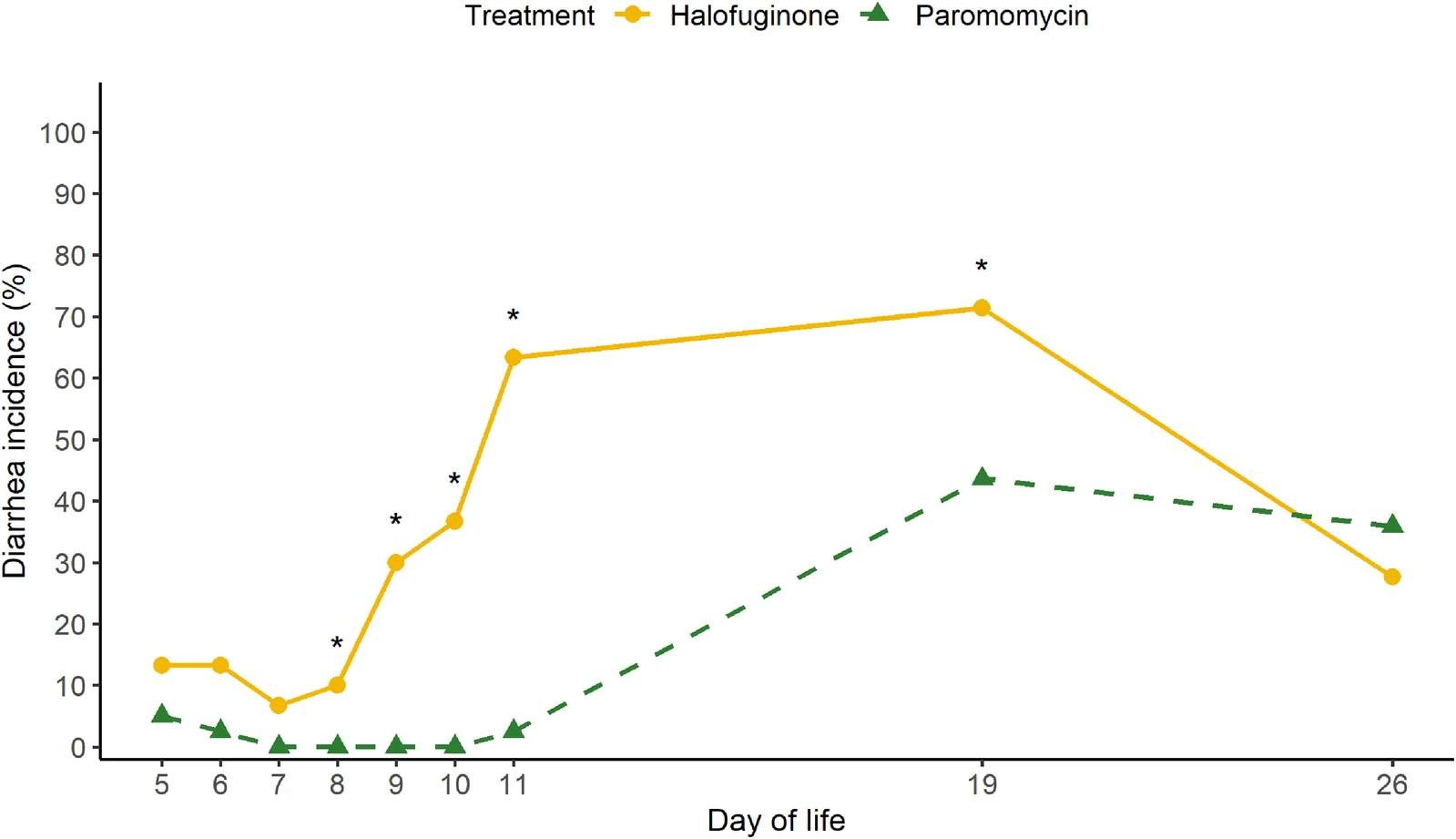
Incidence of diarrhea (fecal score ≥ 2) in dairy calves treated with Paromomycin (n = 40) or Halofuginone (n = 30) across the experimental period. Data are expressed as the percentage of calves presenting diarrhea on each day of life (5, 6, 7, 8, 9, 10, 11, 19, and 26). Asterisks (*) indicate significant differences between treatments at a given *day 8 P = 0.041; day 9 P <0.001; day 10 P <0.0001; day 11 P <0.0001; day 19 P = 0.024)*, as determined by Fisher’s exact test when expected cell counts were <5, or by the chi-square test otherwise.

The temporal pattern of diarrhea incidence differed markedly between treatments (Fig. 1). Calves treated with halofuginone showed an early and pronounced increase in diarrhea incidence beginning on day 8 of life, with a sharp rise between days 9 and 11, when incidence exceeded 60%. Peak incidence was observed on day 19, reaching approximately 70%, followed by a decline by day 26. In contrast, calves treated with paromomycin exhibited consistently lower diarrhea incidence during the first two weeks of life, remaining near zero until day 10. A moderate increase was observed thereafter, with a peak at day 19 (∼40%), which remained substantially lower than that observed in the halofuginone group. Significant differences between treatments were detected at multiple time points (*P* < 0.05), indicating a reduced and delayed diarrhea burden in paromomycin-treated calves.

Because diarrhea was analyzed as a binary outcome (presence/absence) with repeated measurements within the same calf over time, a generalized estimating equation (GEE) model with a logit link function was applied to account for within-calf correlation. The model revealed a significant effect of treatment (P < 0.0001), with calves treated with halofuginone showing substantially higher odds of diarrhea compared with those treated with paromomycin (β= 1.595; odds ratio= 4.93; 95% CI= 2.98–8.17). Day of life also had a significant effect on diarrhea occurrence (P < 0.0001). Across the experimental period, calves receiving halofuginone had approximately fivefold greater odds of experiencing diarrhea than calves treated with paromomycin.

### Days with diarrhea

**Fig. 2:**
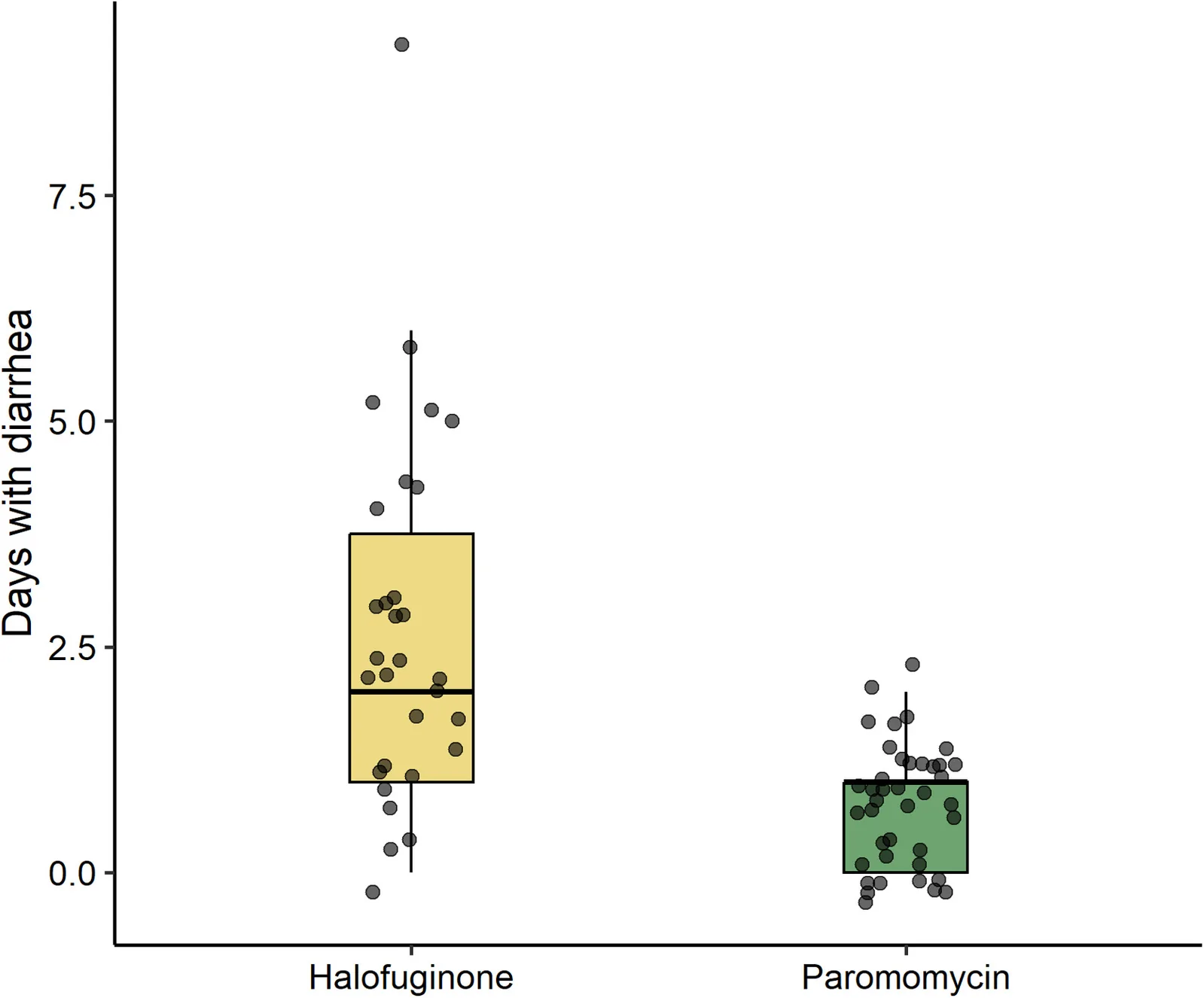
Days with diarrhea in calves treated with Paromomycin (n = 40) or Halofuginone (n = 30). Distribution of the total number of days with diarrhea in calves treated with Halofuginone or Paromomycin. Each dot represents an individual calf. Boxes indicate the median and interquartile range, with whiskers representing data dispersion (*P* < 0.001).

The total number of days with diarrhea per calf was significantly lower in the paromomycin group than in the halofuginone group (Fig. 2). Calves treated with halofuginone had a median of 2.0 days with diarrhea (IQR: 1.0–3.8), whereas calves receiving paromomycin had a median of 0.9 days (IQR: 0.0–1.0) (P < 0.001). The distribution of diarrheic days was also more variable in the halofuginone group, ranging from 0 to 9 days, while paromomycin-treated calves experienced a narrower range of 0 to 2.3 days. Results from the negative binomial regression model confirmed this difference, showing that calves receiving halofuginone experienced 3.4 times more diarrheic days than those treated with paromomycin (rate ratio [RR]= 3.40; 95% CI: 2.20–5.24; P < 0.0001). Notably, most calves in the paromomycin group experienced either no diarrhea or only short-lasting episodes (≤1 day), whereas prolonged diarrheic periods were more frequently observed among calves receiving halofuginone. These findings demonstrate that paromomycin substantially reduced both the duration and overall burden of neonatal diarrhea.

### Days requiring hydration therapy

**Fig. 3:**
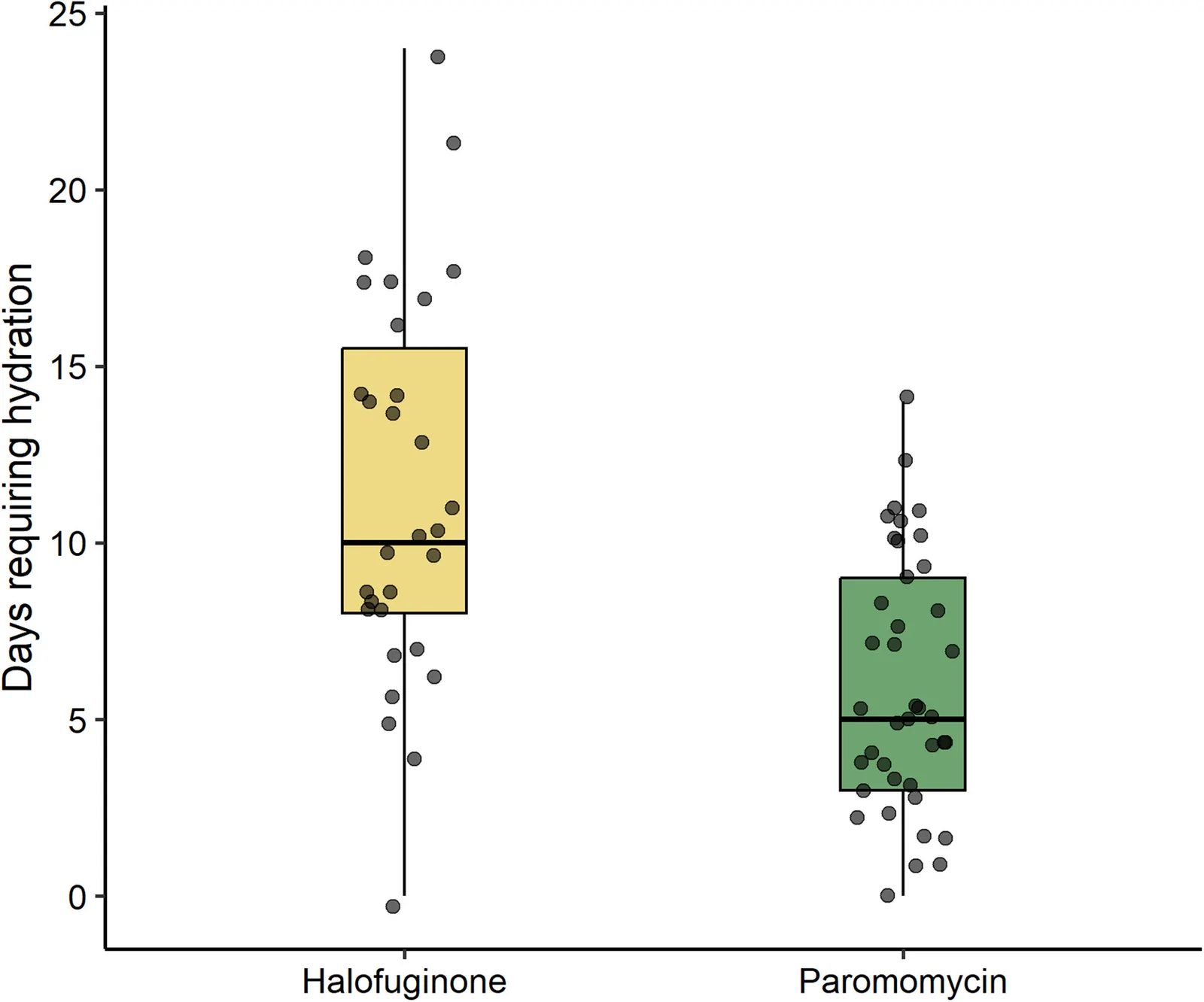
Days requiring hydration in calves treated with Paromomycin (n = 40) or Halofuginone (n = 30). Distribution of the total number of days requiring hydration in calves treated with Halofuginone or Paromomycin. Each dot represents an individual calf. Boxes indicate the median and interquartile range, with whiskers representing data dispersion. *P* < 0.001.

Consistent with the diarrhea outcomes, the number of days requiring oral or parenteral hydration was significantly higher in calves treated with halofuginone than in those treated with paromomycin (Fig. 3). The halofuginone group displayed a higher median number of hydration days and a wider distribution, reflecting more severe or prolonged clinical dehydration. In contrast, paromomycin-treated calves required fewer hydration interventions, with most animals clustering at low values. These results demonstrate that paromomycin treatment was associated with reduced clinical severity of diarrhea, as reflected by a lower need for supportive hydration therapy.

Using negative binomial regression, treatment had a significant effect on the duration of diarrhea and the need for hydration (*P* < 0.0001 for both outcomes). Calves treated with halofuginone experienced a significantly longer duration of diarrhea compared with calves treated with paromomycin (rate ratio [RR]= 3.40; 95% CI= 2.20–5.24), indicating that halofuginone-treated calves had more than three times as many days with diarrhea. In addition, halofuginone treatment was associated with a greater number of days requiring oral hydration (RR= 1.93; 95% CI= 1.48–2.50), corresponding to nearly twice as many hydration days compared with the paromomycin group.

### Economic costs

**Fig. 4:**
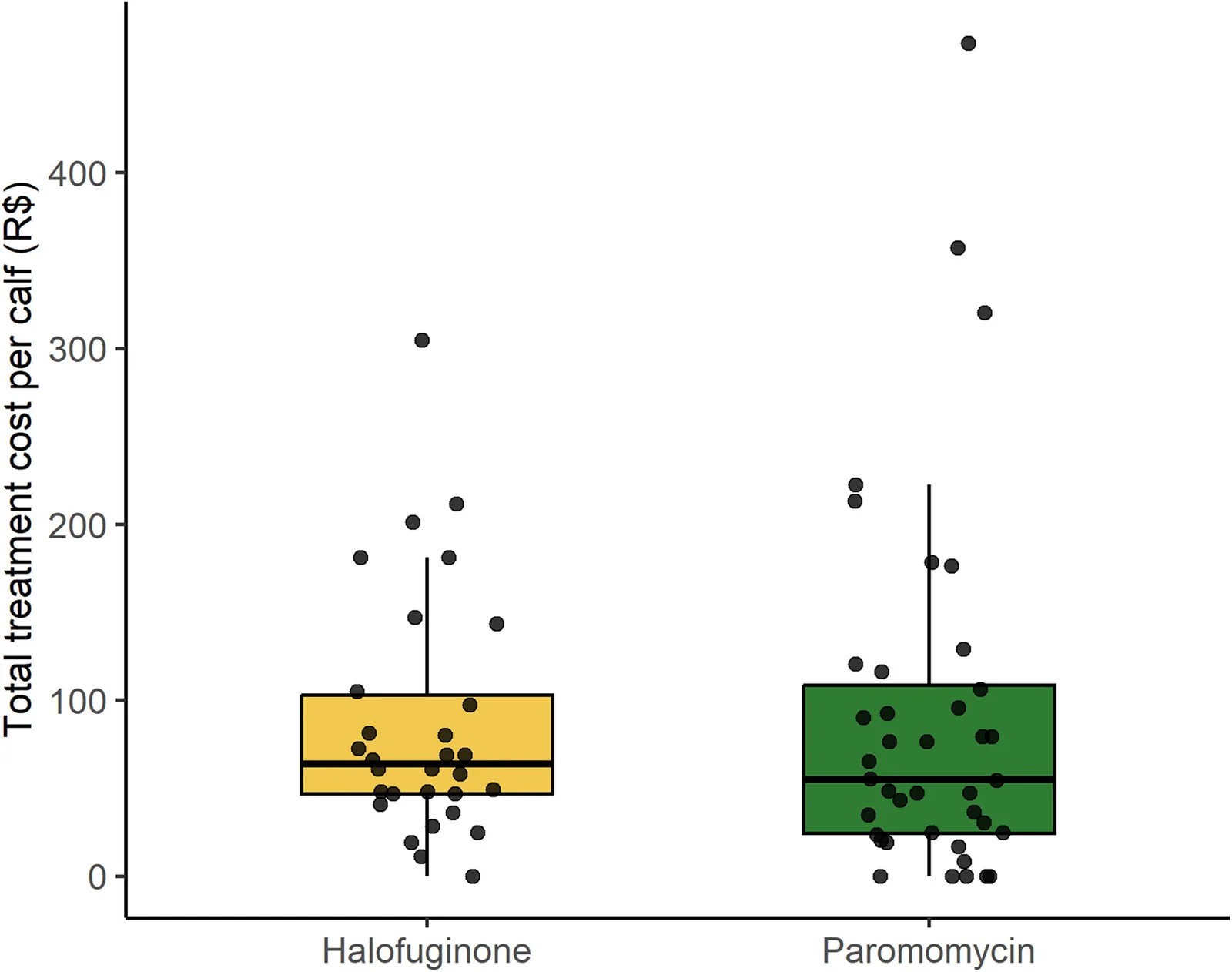
Distribution of hydration cost per calf (R$) in calves treated with Paromomycin (n = 40) or Halofuginone (n = 30). Boxes indicate the interquartile range, horizontal lines represent the median, and dots correspond to individual calves.

Hydration costs did not differ significantly between treatment groups (P = 0.0744; Fig. 4). Costs were estimated using the average commercial price of Altalyte® (Alta Cria) practiced at the farm during the experimental period. Variability in hydration costs was observed within both treatment groups.

### Body weight development

**Fig. 5:**
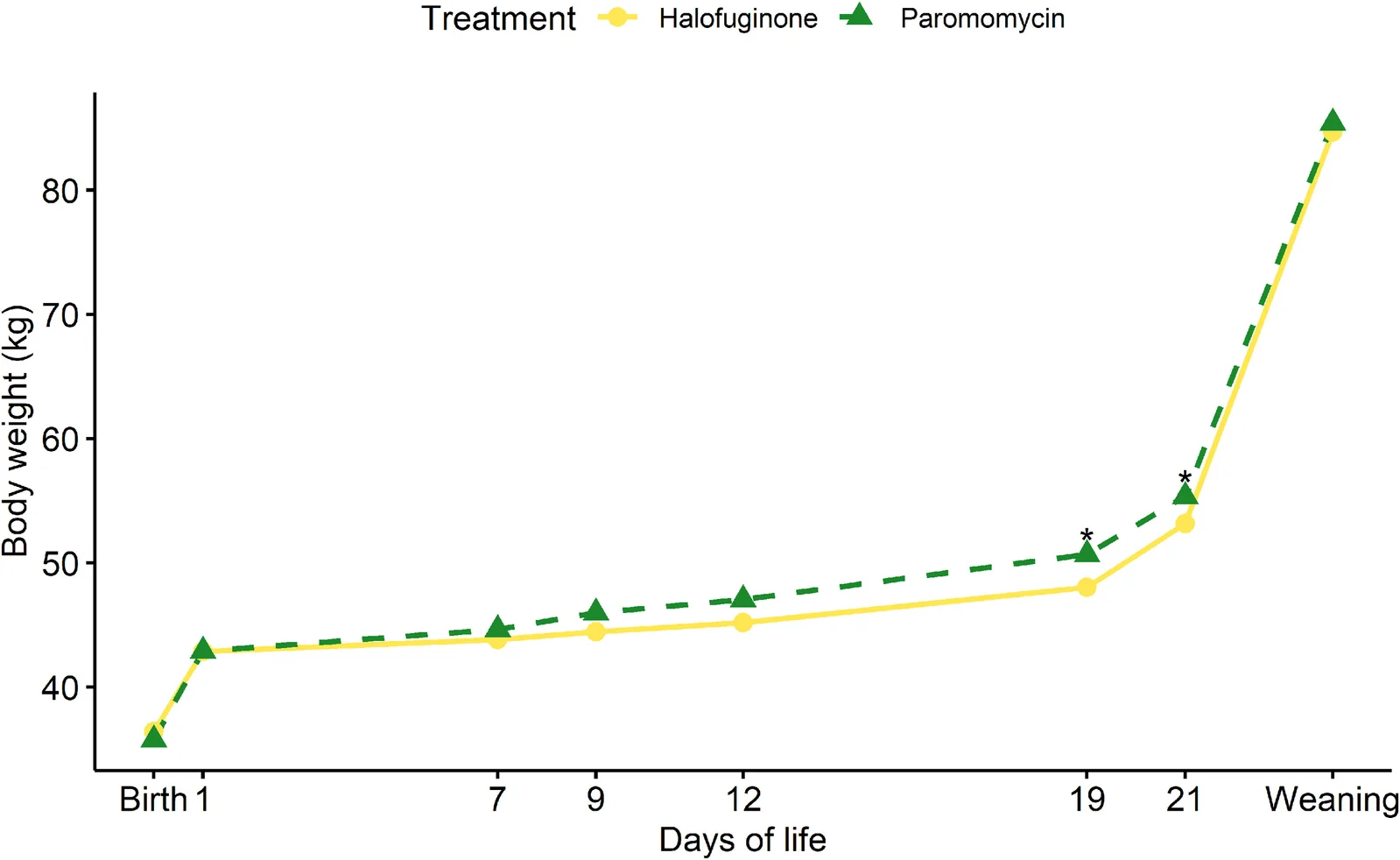
Body weight (kg) of dairy calves treated with Paromomycin (n = 40) or Halofuginone (n = 30) from birth to 21 days of life. Data represent least squares means (LSMeans) estimated using a linear mixed-effects model including treatment, time (days of life), and their interaction as fixed effects, with calf as a random effect (*treatment: P* = 0.2358; *time: P* < 0.0001; *treatment × time: P* < 0.0001). Asterisks (*) indicate days of life with significant differences between treatments based on slice analysis day 14 *P* = 0.0183 and day 21 *P* = 0.0509**.**

Body weight at the start of treatment (Day 5 of life) was similar between groups, averaging 42.9 ± 4.0 kg in the paromomycin group and 42.9 ± 3.4 kg in the halofuginone group (P = 0.970). Body weight increased throughout the study period in both groups (time effect: P < 0.0001; Fig. 5). Although the overall treatment effect was not significant (P = 0.2358), a significant treatment × time interaction was detected (P < 0.0001), indicating that growth patterns differed between treatments over time. Slice analysis identified greater body weight in calves treated with paromomycin at day 14 (P = 0.0183) and day 21 (P = 0.0509). No significant differences were observed between groups at birth, treatment initiation, or weaning.

According to a mixed-effects model, in the paromomycin group, height of withers increased from 75.36 ± 3.62 cm at birth to 89.60 ± 3.74 cm at weaning, while hip width increased from 17.46 ± 2.13 cm to 22.40 ± 1.14 cm. Similarly, calves in the halofuginone group showed an increase in withers height from 75.30 ± 3.02 cm at birth to 88.87 ± 2.76 cm at weaning, and hip width increased from 18.02 ± 1.80 cm to 22.45 ± 0.83 cm. Growth from birth to weaning, expressed as individual change (Δ), did not differ between treatment groups. Increases in withers height were comparable between paromomycin and halofuginone calves (14.24 ± 2.58 vs. 13.57 ± 2.67 cm; *P* = 0.296). Similarly, hip width growth did not differ between groups (4.94 ± 2.12 vs. 4.43 ± 1.86 cm; *P* = 0.295), indicating that skeletal growth trajectories were equivalent regardless of treatment.

### Fecal oocyst excretion

**Fig. 6:**
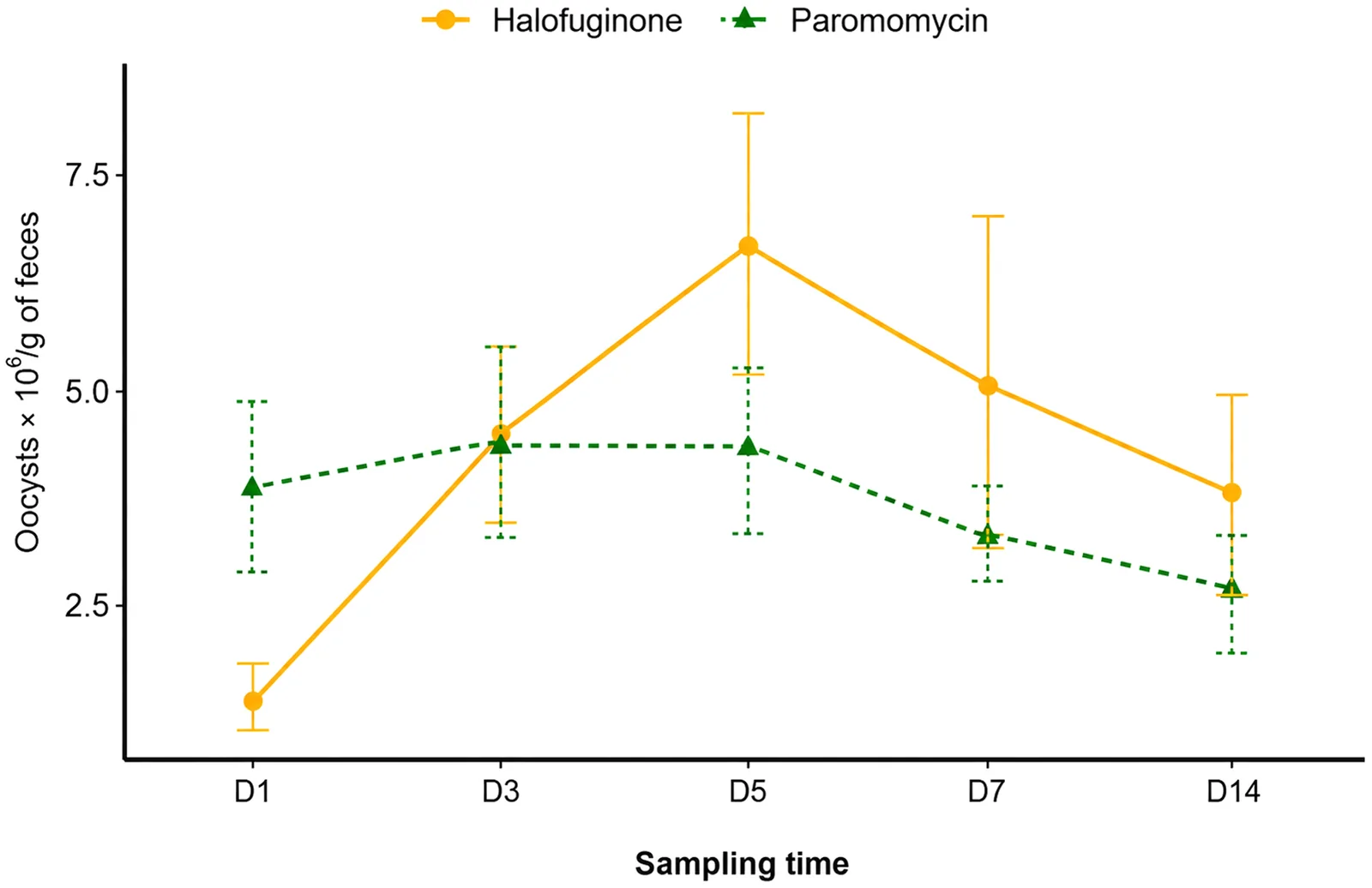
Fecal *Cryptosporidium parvum* oocyst counts in dairy calves treated with Paromomycin (n = 40) or Halofuginone (n = 30). Values are presented as mean ± SEM. Sampling was performed on D1 (5th day of life; immediately before treatment initiation, baseline), D3 (7th day of life; third day of treatment), D5 (9th day of life; fifth day of treatment), D7 (11th day of life; last day of treatment), and D14 (19th day of life; 7 days after the end of treatment).

The temporal pattern of fecal oocyst excretion differed between treatments (Fig. 6). Baseline oocyst counts were assessed on day 1 of the study (D1), corresponding to the fifth day of life and immediately before treatment initiation. Calves treated with halofuginone showed a marked increase in oocyst shedding from D1 to D5, reaching the highest mean excretion at D5, followed by a gradual decline at D7 and D14. In contrast, calves treated with paromomycin exhibited a more stable oocyst excretion profile throughout the evaluation period, with only a slight increase between D1 and D5 and a consistent reduction thereafter.

At all evaluated time points, oocyst excretion tended to be lower and less variable in the paromomycin group than in the halofuginone group, particularly at D5 and D7, when halofuginone-treated calves exhibited higher mean values and greater dispersion. By D14, oocyst shedding had declined in both groups; however, calves receiving paromomycin maintained numerically lower oocyst counts.

## Discussion

The absence of differences in colostrum quality and passive transfer of immunity between treatment groups indicates that both paromomycin- and halofuginone-treated calves originated from a well-managed colostrum program, minimizing the potential confounding effect of colostrum management on health outcomes. Median colostrum Brix values in both groups were well above the threshold commonly associated with high-quality colostrum, and serum Brix values at 48 h of life exceeded the cutoff for failure of passive transfer, reflecting successful immunoglobulin absorption. These findings are consistent with the principles outlined by Godden et al. (2019), who emphasize that early provision of an adequate volume of high-quality colostrum is the single most critical factor determining neonatal calf health and survival. Moreover, the comparable serum Brix values observed between groups align with the classification proposed by Godden et al. (2019), in which serum Brix values ≥8.1% correspond to at least fair passive transfer, with progressively lower morbidity risk as immunoglobulin concentrations increase. By using colostrum and serum Brix as inclusion criteria, the present study ensured that calves entered the trial with similar immune status, thereby strengthening the internal validity of comparisons between treatments. This methodological approach is particularly important because inadequate passive transfer is independently associated with an increased risk of neonatal diarrhea, impaired growth, and long-term reductions in productivity. Therefore, differences in early-life immune status were unlikely to have substantially influenced the clinical outcomes observed in this study.

An important finding of the present study was the unequal distribution of maternal parity between treatment groups, with a greater proportion of calves born to primiparous cows in the paromomycin group. Maternal parity has been associated with variation in colostrum quality, passive transfer, birth weight, and neonatal calf health, with multiparous cows generally producing colostrum with higher immunoglobulin concentrations than primiparous cows (Aydogdu & Guzelbektes, 2018; Silva et al., 2024). Therefore, this imbalance represented a potential source of confounding that warranted further evaluation. To address this concern, colostrum quality was compared between primiparous and multiparous cows within the paromomycin-treated group. No significant difference in colostrum Brix was detected according to maternal parity (P = 0.243). Furthermore, colostrum quality and serum Brix at 48 h of life were comparable between treatment groups, indicating similar passive transfer of immunity at enrollment. Together, these findings suggest that the observed imbalance in maternal parity was not accompanied by measurable differences in colostrum quality or passive immunity. Nevertheless, because only three multiparous cows were included in the paromomycin group, this comparison had limited statistical power and should be interpreted with caution. Consequently, although maternal parity is unlikely to fully explain the differences observed between treatments, its unequal distribution should be recognized as a limitation of the study.

The present findings suggest that paromomycin treatment was associated with a lower incidence and severity of neonatal diarrhea than halofuginone under the conditions of this field study. Although maternal parity was unevenly distributed between treatment groups, the absence of detectable differences in colostrum quality and passive transfer between groups, together with the within-group analysis according to parity, reduces the likelihood that the observed clinical differences were primarily driven by maternal factors. Nevertheless, the unequal distribution of maternal parity should be considered when interpreting the results. Adequate passive transfer has consistently been shown to reduce overall morbidity but does not prevent cryptosporidiosis, which relies predominantly on local intestinal immunity and epithelial integrity rather than systemic antibodies (Adkins, 2022).

The comparison between paromomycin and halofuginone is particularly relevant because these compounds currently represent the two main oral interventions with demonstrated anticryptosporidial activity under field conditions in neonatal calves (Almawly et al., 2013; Bellosa et al., 2011; De Waele et al., 2010; Fayer & Ellis, 1993; Grinberg et al., 2002; Niine et al., 2018; Silverlås et al., 2009; Trotz-Williams et al., 2011). Halofuginone has long been the only licensed product for the prevention of bovine cryptosporidiosis and remains widely used in commercial dairy systems. However, concerns regarding its narrow therapeutic window and the potential occurrence of adverse effects, including reduced milk intake, anorexia, and intestinal irritation, have stimulated the search for alternative strategies capable of controlling cryptosporidiosis while preserving calf performance (Bernal-Córdoba et al., 2025; Brainard et al., 2020). In this context, the superior clinical outcomes observed in paromomycin-treated calves are particularly noteworthy because they were achieved under commercial conditions characterized by a high challenge of neonatal diarrhea, thereby supporting previous evidence that paromomycin may provide effective control of cryptosporidiosis while minimizing negative impacts on calf growth and welfare.

### Diarrhea dynamics and clinical severity

Calves treated with halofuginone exhibited an early and pronounced increase in diarrhea incidence, particularly between days 8 and 11 of life, coinciding with the expected peak of Cryptosporidium parvum infection and oocyst shedding in neonatal calves. This temporal pattern is consistent with the established pathogenesis of cryptosporidiosis, in which epithelial invasion, villous atrophy, and crypt hyperplasia result in malabsorptive diarrhea typically manifesting during the second week of life (Adkins, 2022). In contrast, paromomycin-treated calves showed a delayed and attenuated diarrhea profile, remaining largely free of clinical signs during the first 10 days of life and experiencing substantially fewer diarrheic days overall.

The GEE analysis confirmed these observations, indicating that halofuginone-treated calves had nearly fivefold greater odds of developing diarrhea compared with paromomycin-treated calves. These findings align with growing evidence that, although halofuginone can reduce oocyst shedding under certain conditions, its effects on clinical diarrhea and calf comfort are inconsistent and highly dependent on timing, dose, and management conditions (Brainard et al., 2020). Moreover, previous studies have reported that halofuginone-treated calves frequently experience persistent or recurrent diarrhea despite reductions in parasite burden, suggesting a dissociation between parasitological and clinical outcomes (Vélez et al., 2019).

### Duration of diarrhea and hydration requirements

Beyond incidence, the duration of diarrhea and need for hydration therapy are critical indicators of disease severity and animal welfare. In the present study, halofuginone-treated calves experienced more than three times as many diarrheic days and nearly twice as many days requiring hydration compared with calves receiving paromomycin. These findings are clinically relevant, as prolonged diarrhea exacerbates dehydration, electrolyte imbalances, and negative energy balance, all of which compromise growth and increase labor and treatment costs.

The reduced need for hydration in paromomycin-treated calves suggests improved intestinal function and fluid absorption, likely reflecting better preservation of epithelial integrity. This interpretation is supported by previous work demonstrating that paromomycin reduces C. parvum replication within enterocytes while allowing controlled antigen exposure and the development of local immunity, resulting in fewer and milder clinical signs (Vasquez-Flores et al., 2024). In contrast, halofuginone has been described as having a narrow therapeutic window and potential toxic effects on intestinal and systemic metabolism, which may contribute to prolonged clinical recovery despite partial parasite suppression (Brainard et al., 2020).

### Fecal oocyst excretion patterns

Differences in fecal oocyst shedding further support the superior clinical performance of paromomycin. Halofuginone-treated calves exhibited a pronounced mid-period peak in oocyst excretion, particularly at day 5, accompanied by substantial interindividual variability. In contrast, paromomycin-treated calves showed a more stable and controlled shedding profile across time points, with lower mean values and reduced dispersion.

This pattern is consistent with previous field trials demonstrating that paromomycin can reduce oocyst shedding by 34–50% during the peak of cryptosporidiosis while avoiding the rebound shedding sometimes observed following halofuginone treatment (Vasquez-Flores et al., 2024). Importantly, high oocyst shedding has been strongly associated with both increased diarrhea severity and environmental contamination, amplifying infection pressure within calf-rearing facilities (Adkins, 2022).

Therefore, the more controlled shedding observed with paromomycin may have implications not only for individual calf health but also for herd-level disease dynamics. The relationship between fecal consistency and oocyst shedding observed in this study is further supported by experimental evidence showing that higher fecal scores are closely associated with lower fecal dry matter and increased C. parvum oocyst excretion (Bellosa et al., 2011). Thus, the reduced diarrhea burden in paromomycin-treated calves is biologically consistent with their lower and more uniform oocyst output.

### Growth performance and long-term implications

Improved growth performance in paromomycin-treated calves provides additional evidence of the clinical relevance of these findings. From the second week of life onward, calves receiving paromomycin exhibited greater body weight, with significant differences observed at days 19 and 21 and persisting through weaning. Early-life growth depression associated with neonatal diarrhea has been repeatedly linked to delayed age at first calving, reduced milk yield in the first lactation, and increased rearing costs (Boulton et al., 2017). Previous studies evaluating halofuginone have reported neutral or even negative effects on early weight gain, despite reductions in parasite detection (Vélez et al., 2019). In contrast, paromomycin has consistently been associated with improved growth trajectories under field conditions, particularly when administered during the early window of cryptosporidial challenge (Vasquez-Flores et al., 2024). The superior growth observed in the present study is therefore likely a direct consequence of reduced diarrhea severity, shorter disease duration, and improved nutrient utilization.

Importantly, the implications of neonatal diarrhea extend beyond the preweaning phase and may influence lifetime productivity. In a large retrospective cohort including 1907 Holstein cows, Hou et al. (2026) demonstrated that calves experiencing neonatal diarrhea exhibited persistent reductions in lactation performance across multiple parities, including lower 305-d milk yield, reduced peak milk production, and impaired milk composition. Notably, these negative effects accumulated over time and remained evident even in later parities, reinforcing the concept that early-life gastrointestinal disturbances can permanently alter developmental trajectories and productive potential. In this context, the marked reduction in diarrhea incidence and duration observed in calves receiving paromomycin in the present study is likely to translate into long-term benefits at the herd level. Therefore, interventions targeting enteric health in the neonatal period should be interpreted not only as short-term clinical strategies but also as key drivers of future lactation efficiency and economic sustainability in dairy systems.

The growth responses observed in the present study are also consistent with previous field observations reported by Vázquez-Flores et al. (2024), who demonstrated that calves receiving paromomycin (50 mg/kg for 7 days) exhibited lower diarrhea incidence and oocyst excretion together with greater body weight at 60, 90, and 120 days of age compared with calves treated with halofuginone. Although the present study detected significant differences primarily at days 14 and 21 of life, the direction of the response was remarkably similar, suggesting that the early reduction in enteric disease burden may have positive consequences for subsequent growth. Collectively, these findings strengthen the evidence supporting paromomycin as an effective alternative to halofuginone for the control of neonatal cryptosporidiosis under commercial dairy conditions.

### Economic considerations

Although total treatment costs did not differ statistically between groups, the descriptive analysis revealed a trend toward higher and more variable costs in halofuginone-treated calves. This variability reflects the strong influence of individual clinical evolution, particularly prolonged diarrhea and repeated hydration therapy, on overall expenses. From an economic standpoint, even modest increases in early-life morbidity can substantially impact the cost of rearing replacement heifers, given the cumulative effects on labor, treatments, and growth efficiency (Boulton et al., 2017). Taken together, these findings suggest that paromomycin offers a more favorable balance between clinical efficacy, growth performance, and economic sustainability. By reducing diarrhea burden without exacerbating clinical severity or growth suppression, paromomycin may represent a more suitable therapeutic option for the management of neonatal cryptosporidiosis under commercial dairy conditions.

## Conclusion

In conclusion, paromomycin treatment resulted in lower diarrhea incidence, shorter disease duration, reduced need for supportive therapy, improved growth, and a more controlled oocyst shedding pattern compared with halofuginone. These advantages were observed despite equivalent colostrum management and passive immunity, underscoring the robustness of the treatment effect. The present findings reinforce growing concerns regarding the clinical limitations of halofuginone and support paromomycin as an effective and biologically coherent alternative for the control of cryptosporidiosis in neonatal calves.

## Ethics declaration

### Animal subject

This study was conducted in accordance with the ARRIVE (Animal Research: Reporting of In Vivo Experiments) guidelines.

This study was approved by the All procedures involving animals were approved by the Ethics Committee on Animal Use (CEUA) of the School of Veterinary Medicine and Animal Science, University of São Paulo (FMVZ-USP), São Paulo, Brazil, under protocol no. 2614050526. The study was conducted in accordance with the ARRIVE guidelines and applicable institutional and national regulations for animal welfare. (Approval No. 2614050526)

### Ethics statement

The experimental protocols used in this study were approved by the Ethics Committee on Animal Use of the School of Veterinary Medicine and Animal Science of the University of São Paulo (protocol number: 1421110729).

### Declaration of generative AI and AI-assisted technologies in the manuscript preparation process

During the preparation of this work the author(s) used ChatGPT to correct the English language. After using this tool/service, the author(s) reviewed and edited the content as needed and take(s) full responsibility for the content of the published article.

## CRediT authorship contribution statement

**B.P. Santarosa:** Writing – original draft, Validation, Supervision, Project administration, Methodology, Investigation, Funding acquisition, Formal analysis, Data curation, Conceptualization. **M.P.D.F. Branco:** Resources, Investigation, Funding acquisition. **G.L. Marques:** Project administration, Investigation, Funding acquisition. **K.N. da Silva:** Writing – review & editing, Methodology. **A.L. Lelis:** Writing – review & editing, Visualization, Validation, Methodology. **G.M. Januario:** Methodology, Investigation, Data curation. **G.G. Silveira:** Methodology, Investigation, Formal analysis, Data curation. **R.E. Lopes:** Writing – review & editing, Validation, Supervision, Resources, Project administration.

## Declaration of competing interest

The authors declare the following financial interests/personal relationships which may be considered as potential competing interests:

Bianca Paola Santarosa reports was provided by Huvepharma NV. Gabriella Macedo Januario reports financial support was provided by State of Sao Paulo Research Foundation. Bianca Paola Santarosa reports a relationship with Huvepharma NV that includes: employment. The authors have no additional relationships or activities to declare beyond those already disclosed in the Conflict of Interest statement. If there are other authors, they declare that they have no known competing financial interests or personal relationships that could have appeared to influence the work reported in this paper.

## Appendix Supplementary materials

Image, application 1
