# Supplementary material for: Paromomycin improves clinical outcomes and growth performance compared with halofuginone in dairy calves naturally exposed to Cryptosporidium parvum
Source: Vet Anim Sci. 2026 Aug 18;33:100828. doi: 10.1016/j.vas.2026.100828 (PMC13543089; doi:10.1016/j.vas.2026.100828)
Supplement: Supplementary file 1 [file mmc1.pdf]

CERTIFICADO

Certificamos que a proposta intitulada "Prevenção da Criptosporidiose em Neonatos Bovinos: Estudo Comparativo dos Efeitos de Halofuginona e Paromomicina sobre Indicadores Clínicos, Parasitológicos e Microbiota Fecal", protocolada sob o CEUA nº 2614050526 (ID 012045), sob a responsabilidade de **Fábio Celidonio Pogliani** *e equipe; Gabriella de Macedo Januário* - que envolve a produção, manutenção e/ou utilização de animais pertencentes ao filo Chordata, subfilo Vertebrata (exceto o homem), para fins de pesquisa científica ou ensino - está de acordo com os preceitos da Lei 11.794 de 8 de outubro de 2008, com o Decreto 6.899 de 15 de julho de 2009, bem como com as normas editadas pelo Conselho Nacional de Controle da Experimentação Animal (CONCEA), e foi **APROVADA** pela Comissão de Ética no Uso de Animais da Faculdade de Medicina Veterinária e Zootecnia Universidade de São Paulo (CEUA/FMVZ) na reunião de 01/07/2026.

We certify that the proposal "Prevention of Cryptosporidiosis in Bovine Neonates: A Comparative Study of the Effects of Halofuginone and Paromomycin on Clinical, Parasitological and Fecal Microbiota Indicators", utilizing 60 Bovines (60 females), protocol number CEUA 2614050526 (ID 012045), under the responsibility of **Fábio Celidonio Pogliani** *and team; Gabriella de Macedo Januário* - which involves the production, maintenance and/or use of animals belonging to the phylum Chordata, subphylum Vertebrata (except human beings), for scientific research purposes or teaching - is in accordance with Law 11.794 of October 8, 2008, Decree 6899 of July 15, 2009, as well as with the rules issued by the National Council for Control of Animal Experimentation (CONCEA), and was **APPROVED** by the Ethic Committee on Animal Use of the School of Veterinary Medicine and Animal Science (University of São Paulo) (CEUA/FMVZ) in the meeting of 07/01/2026.

Finalidade da Proposta: Pesquisa

Vigência da Proposta: de 07/2026 a 06/2027   Área: Clínica Médica Veterinária

Origem:	Animais provenientes de estabelecimentos comerciais						
Espécie:	Bovinos	sexo:	Fêmeas	idade:	5 a 75 dias	Quantidade:	60
Linhagem:	Holandesa			Peso:	25 a 140 kg		

São Paulo, 22 de julho de 2026

Prof. Dr. Marcelo Bahia Labruna
Coordenador da Comissão de Ética no Uso de Animais
Faculdade de Medicina Veterinária e Zootecnia
Universidade de São Paulo

Prof. Dr. Marcelo Demarchi Goissis
Vice-Coordenador da Comissão de Ética no Uso de Animais
Faculdade de Medicina Veterinária e Zootecnia
Universidade de São Paulo
